# Chronic persistent Horner’s syndrome in trigeminal autonomic cephalalgia subtypes and alleviation with treatment: two case reports

**DOI:** 10.1186/s13256-019-1986-y

**Published:** 2019-03-14

**Authors:** Todd D. Rozen, Matthew T. Kline

**Affiliations:** 1 Mayo Clinic Florida, Jacksonville, 4500 San Pablo Road, Jacksonville, FL 32224 USA; 2 Private Practice Interventional Pain Management, Willow Grove, PA USA

**Keywords:** Trigeminal autonomic cephalalgia, Horner’s syndrome, Ptosis, Hemicrania continua, LASH syndrome

## Abstract

**Background:**

The trigeminal autonomic cephalalgias are a group of primary headache syndromes marked by severe head pain and associated cranial autonomic symptoms which can include a full or partial Horner’s syndrome. Rarely, the eye-related symptoms will become fixed even between headache attacks. There is minimal documentation that the Horner’s syndrome can be reversed if successful treatment of the underlying headache disorder is initiated.

**Case reports:**

Two cases are presented of trigeminal autonomic cephalalgia subtypes with chronic persistent Horner’s syndromes that alleviated with treatment of the underlying primary headache disorder. Patient 1, an 82-year-old Caucasian woman, presented with hemicrania continua with a partial Horner’s syndrome that was present for 2 years. She was unable to take indomethacin as she was on anticoagulation. After a C2–3 diagnostic facet injection, not only did she become pain free but her ptosis completely resolved. She then underwent a radiofrequency facet neurotomy with complete alleviation of head pain and complete resolution of her ptosis. Patient 2, a 21-year-old Caucasian woman, presented with long-lasting autonomic symptoms with hemicrania syndrome and a fixed miosis and ptosis of 6 months’ duration. After achieving 2 months of pain freedom on indomethacin her Horner’s syndrome completely resolved.

**Conclusion:**

A chronic fixed partial or full Horner’s syndrome can occur in trigeminal autonomic cephalalgia subtypes, but it can also be reversed in patients with treatment even after months to years of duration. This would suggest that the sympathetic dysfunction leading to the eye-related symptoms is from irritation of the sympathetic chain rather than permanent injury as the result of vasodilatory trauma after trigeminal autonomic reflex activation.

## Background

The trigeminal autonomic cephalalgias (TACs) are a group of primary headache syndromes marked by severe head pain and associated cranial autonomic symptoms which can include a full or partial Horner’s syndrome [1]. Rarely, the eye-related symptoms will become fixed even between headache attacks [2–7]. There are minimal to no data in the medical literature stating whether Horner’s syndrome can be reversed with effective treatment of the underlying headache disorder. Two cases are presented of complete alleviation of a persistent full and partial Horner’s syndrome with treatment of the TAC syndrome.

## Case presentations

### Case 1

An 82-year-old Caucasian woman presented to a headache specialty clinic with a 2.5 year history of daily persistent left-sided headaches. The age of headache onset was 79 years. She had a previous history of migraine without aura, also left sided, which would occur approximately once per month. Her headache may have started as a daily persistent headache from onset or become daily over a short period of time. She could not exactly define the temporal profile of onset. The pain location was entire left hemicranium from periorbit/retro-orbit to occipitonuchal region with the forehead and temple being the most significant area for pain. Her average daily pain intensity was 8/10 on a visual analog scale (VAS) and she would also experience exacerbations to 10/10 several times per week and these peak pain periods would last from hours to 1 full day. During the peaks she would develop migrainous (nausea, vomiting, photophobia, and phonophobia) and cranial autonomic symptoms (eyelid ptosis, lacrimation) as well as agitation. Her prior migraines never included any cranial autonomic issues. Very early on in the course of the headaches she developed a left-sided ptosis during a period of pain exacerbation that never resolved. The ptosis was present for at least 2 years at the time of her consultation.

Her past medical history was marked by several concussions during her teens and several whiplash injuries as an adult with resultant neck pain. She had atrial fibrillation and was on chronic anticoagulation therapy. She had hyperlipidemia and ulcerative colitis. Her past surgical history was marked by a cervical spine fusion from C3–7. She was a chronic tobacco smoker × 50 years. Her family history was negative including no headache disorders.

Prior to coming for consultation she had tried and failed various preventive medications including gabapentin (200 mg), valproic acid (1250 mg), amitriptyline (50 mg), nortriptyline (50 mg), propranolol (80 mg), daily oxycodone (10–15 mg per day), and onabotulinum toxin A injections. At the time of presentation she was on no headache preventive medication. She was using daily acetaminophen/aspirin/caffeine tablets up to eight per day as well as injectable sumatriptan up to 4 days per week. Her other daily non-headache-related medications included: apixaban (5 mg twice a day) and darifenacin hydrobromide (15 mg/day).

On examination she was afebrile, normotensive (118/69 mmHg), with a pulse rate of 60 beats per minute (bpm). A general examination was non-focal. Her neurologic/headache examination was abnormal with a positive left eyelid ptosis without a miotic pupil. There was no evidence of blepharospasm or facial spasm noted on examination to mimic the ptosis. She had tenderness to palpation over the left greater occipital nerve, left atlantoaxial joint, and the left C2–3 facet. No tenderness was noted over the left supraorbital or trochlear regions. No temporal allodynia was identified and she had positive temporal artery pulses.

Her history was very suggestive of hemicrania continua (HC) but she could not be prescribed orally administered indomethacin as she was on chronic anticoagulation therapy. HC is a primary headache syndrome marked by persistent one-sided head pain, usually of mild intensity, with pain exacerbation periods consisting of severe headache that lasts from hours to days with associated migrainous and cranial autonomic symptoms. It is one of the indomethacin-sensitive headache disorders. An injectable indomethacin (indo-test) could have been utilized to verify the diagnosis but it is not available in the USA. A presumed diagnosis of probable HC was made as she met all *International Classification of Headache Disorders* (ICHD-3) criteria except for indomethacin responsiveness [1]. Based on her cervical examination and past neck traumas with cervical fusion, a secondary form of HC from a cervicogenic generator (head pain originating in the upper cervical spine, most likely from activation of the trigeminocervical complex) was also suggested.

Laboratory testing included a normal erythrocyte sedimentation rate and C-reactive protein (CRP). Neuroimaging including a brain magnetic resonance imaging (MRI) and magnetic resonance (MR) angiography of the intracranial vessels identified a 3 mm left ophthalmic artery aneurysm, but even with surgical repair (coiling) the headaches did not improve. No other secondary issues were noted on imaging including ruling out a carotid dissection with conventional angiography. Pituitary hormones were also tested (prolactin, growth hormone, insulin-like growth factor 1) and were within normal range.

**Fig. 1:**
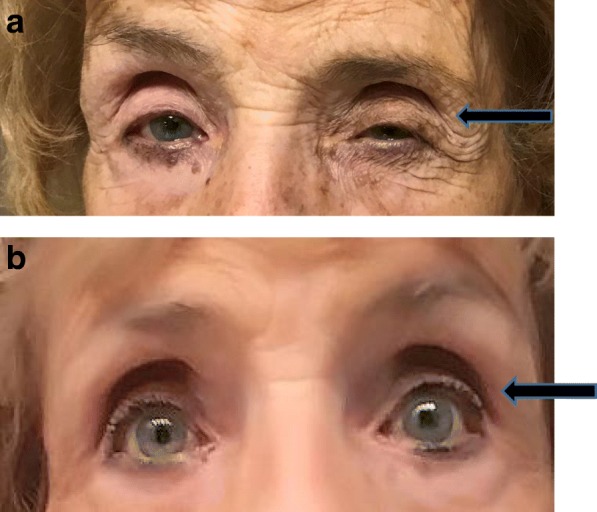
**a** Patient with a trigeminal autonomic cephalalgia variant and a fixed left-sided ptosis (*arrow*). There was no evidence of blepharospasm or facial spasm noted on examination to mimic the ptosis. **b** Complete alleviation of a fixed left-sided ptosis after cervical facet radiofrequency ablation at C2–3 (*arrow*). Image taken 6 weeks after radiofrequency ablation procedure

At her headache consultation she was given a high volume suboccipital nerve block (9 cc of 1% lidocaine and 1 cc of triamcinolone 40 mg/ml) leading to complete pain freedom, but only for 2 days. During this time her ptosis did improve or resolve transiently until her pain returned. As the authors previously reported on alleviation of HC with specific nerve injection procedures, our patient underwent a sphenopalatine block with moderate success [8]. She had no improvement with supraorbital/supratrochlear blocks. An atlantoaxial injection also showed a negative response. She had a positive response to a left C2 dorsal root ganglia (DRG) injection, but a confirmatory procedure did not help. After a C2–3 diagnostic facet injection, not only did she become pain free but her ptosis completely resolved. A confirmatory injection at the same location alleviated her head pain again, thus she underwent a radiofrequency facet neurotomy (a procedure that supplies heat to spinal-based nerves to suppress pain transmission) with complete alleviation of head pain and complete resolution of her ptosis (Fig. 1a, b). After 3.5 months her headache and ptosis returned so a repeat radiofrequency procedure was completed with subsequent resolution of head pain and ptosis through an additional 3-month follow-up.

### Case 2

A 21-year-old Caucasian woman presented for consultation with a 1-year history of headaches. She had no prior history of head pain when she began to develop right-sided only headaches which would last from 2 to 3 days in duration. The headaches were located in a retro-orbital, periorbital, and temporal distribution. The pain was moderate to severe in intensity and would escalate to maximum intensity over approximately 1 hour. Initially the headaches occurred once per week but escalated to two to three times per week. Associated symptoms included migrainous (nausea and rare vomiting, photophobia, phonophobia, and osmophobia) and cranial autonomic features (right eyelid ptosis and miosis, conjunctival injection, lacrimation, nasal congestion, and orbital edema). Her cranial autonomic symptoms would start several hours before headache onset, last the entire duration of the headache and would then outlast the headache for several hours. Six months into her headache history she developed a right-sided full Horner’s syndrome with a fixed ptosis and miosis during a severe headache that never ceased, even between headache attacks.

Her past medical history was marked by a diagnosis of Turner’s syndrome and she also had major depression controlled on medication. She did not smoke tobacco. She was currently a student. Her family history was only significant for migraine in her sister whose headaches lacked any cranial autonomic symptoms. In regard to medications she was taking acetaminophen abortively but had tried no headache preventive medications prior to consultation. She was on sertraline 75 mg for major depression but that was prescribed long before she developed her headaches.

On examination she was normotensive (100/60 mmHg) with normal pulse (72 bpm) and temperature. A general physical examination was normal except for short stature. A neurologic/headache examination (during a headache) demonstrated right-sided head allodynia with right temple, supraorbital, and trochlear nerve tenderness. She had a right-sided miotic pupil with a ptosis. Her neurovascular examination was normal with no supraclavicular, carotid, cranial, or orbital bruits. She also had no greater occipital nerve or upper cervical facet tenderness to palpation.

A diagnosis of long-lasting autonomic symptoms with hemicrania (LASH) was made based on the one-sided nature of the headaches, their episodic presentation, and, most importantly, the temporal profile of onset and offset of her cranial autonomic symptoms [9]. At present there are no ICHD-3 criteria for LASH syndrome although more patients with the disorder are being reported and the present case patient’s headache is consistent with prior documented cases [1, 9]. LASH is considered one of the indomethacin-responsive headaches. The lack of interictal pain in between headache attacks ruled out HC.

Neuroimaging including a brain MRI with pituitary cuts and MR angiography of head and neck vessels with dissection protocol were completed and were normal. Pituitary hormones were also tested (prolactin, growth hormone, insulin-like growth factor 1) and were within normal range.

Short-acting indomethacin was prescribed for LASH syndrome and at a dose of 150 mg per day she was basically pain free with one breakthrough headache per month. At a dose of 200 mg per day she became completely headache free. After achieving 2 months of pain freedom on indomethacin her Horner’s syndrome completely resolved. She was followed-up for another 1 year without headache or miosis/ptosis recurrence, but she was unable to come off indomethacin without her headaches returning.

## Discussion

Two cases are presented of TAC subtypes with chronic persistent Horner’s syndromes that alleviated with treatment of the underlying primary headache disorder. Patient 1 presented with HC with a partial Horner’s syndrome that was present for 2 years. After a C2–3 diagnostic facet injection, not only did she become pain free but her ptosis completely resolved. She then underwent a radiofrequency facet neurotomy with complete alleviation of head pain and complete resolution of her ptosis. Patient 2 presented with LASH syndrome and a fixed miosis and ptosis of 6 months’ duration. After achieving 2 months of pain freedom on indomethacin her Horner’s syndrome completely resolved. These are the first two documented cases in the literature of resolution of a chronic Horner’s syndrome with treatment of the primary headache disorder. The presumed mechanism of a fixed or partial Horner’s syndrome with the TACs is postganglionic cervical sympathetic dysfunction as the result of activation of the trigeminal autonomic reflex [10, 11]. In essence, via a brainstem connection between the trigeminal cervical complex and superior salivatory nucleus (region of cranial parasympathetic outflow), trigeminal nerve stimulation can lead to cranial parasympathetic efferent activation with resulting release of neuropeptides (vasoactive intestinal peptide) which then leads to cranial/extracranial arterial vasodilation [12]. Vasodilation of the internal carotid artery with presumed secondary compression of the oculosympathetic fibers that course along with it and into the cavernous sinus is the suggested etiology of the sympathetic dysfunction [10, 11]. As most TACs are marked by very short-lasting headache attacks (seconds to several hours), not developing a fixed ptosis or miosis would be the predicted norm as the trigeminal autonomic reflex is only activated for brief periods of time, thus any vasodilatory trauma to the cervical sympathetic chain would also be short lived. However, in patients with the chronic form of TACs (chronic cluster headache, chronic paroxysmal hemicrania) or with longer duration severe headache attacks (HC and LASH) or with more frequent daily attacks (short-lasting unilateral neuralgiform headache attacks with conjunctival injection and tearing, which is abbreviated to SUNCT), a permanent sympathetic chain fiber injury could potentially occur. At present there are only rare documented cases of a persistent partial or full Horner’s syndrome in patients with cluster headache from several case series and the case of a single patient with SUNCT has also been reported [2–7]. The two presented case reports are the first to document a persistent and partial Horner’s syndrome with LASH and HC. The fascinating and unexpected resolution of a chronic fixed ptosis and miosis of long duration (6 months and 2 years) would suggest reversible sympathetic dysfunction from irritation of the sympathetic chain rather than permanent injury as the result of vasodilatory trauma. The presumed mechanism as to how the cervical facet radiofrequency procedure helped alleviate the ptosis is suppression of the trigeminal cervical complex at C2 with feedback suppression of the trigeminal autonomic reflex with resolution of internal carotid artery dilation and compression of the sympathetic chain [8]. In regard to how indomethacin was able to alleviate Horner’s syndrome, one would assume this was also by suppressing the trigeminal autonomic reflex. However, the exact mechanism by which indomethacin is able to relieve HC, LASH, and paroxysmal hemicrania is unknown.

## Conclusion

Based on the presented case reports a chronic fixed partial or full Horner’s syndrome can occur in TAC subtypes, but it can also be reversed in patients with treatment even after months to years of duration. This has not been previously documented. Short-term resolution of Horner’s syndrome with diagnostic nerve blocks may be a positive predictive factor for more permanent resolution with longer term anesthesiologic procedures such as radiofrequency ablation.

## Abbreviations

bpm: Beats per minute
CRP: C-reactive protein
DRG: Dorsal root ganglia
HC: Hemicrania continua
ICHD: *International Classification of Headache Disorders*
LASH: Long-lasting autonomic symptoms with hemicrania
MR: Magnetic resonance
SUNCT: Short-lasting unilateral neuralgiform headache attacks with conjunctival injection and tearing
TACs: Trigeminal autonomic cephalalgias
VAS: Visual analog scale

## Authors’ information

There is no relevant information about the authors that may aid in the readers’ interpretation of the article, and understanding of the standpoint of the authors.
